# University of State of Ohio

**Published:** 1890-08

**Authors:** Henry J. Detmers

**Affiliations:** University State of Ohio. Residence, 35 King Avenue, Columbus, Ohio


### VETERINARY COLLEGE NOTES.

*University of State of Ohio. State Appropriations.*—The General Assembly has made an appropriation to the University for equipment of chemical building, $20,000, and for the veterinary building, $5000. Heretofore the veterinary department has been hampered by want of a good hospital and sufficient clinical facilities. This want has been supplied by the recent liberal act of the Legislature.

The new building thus provided for will enable the University to offer to students of veterinary science as good facilities for thorough and practical instruction as are to be found anywhere in America. It will contain lecture rooms, laboratories, a large apartment for the treatment of diseased or injured animals, stalls in which patients needing continued treatment can be kept, and rooms to be occupied by students who will have charge of the hospital day and night.

It is expected that the building will be completed in time to receive patients at the opening of the next University year, September 17th, 1890. After that date patients will be received at any hour. The students of veterinary medicine will thus have abundant opportunities for clinical observation and practice.

Prof. HENRY J. DETMERS, M.D.V.,
*University State of Ohio.*

*Residence, 35 King Avenue, Columbus, Ohio.*

